# Special Issue: Carbon–Refractory Metal Nanostructures: Synthesis, Characterization and Applications

**DOI:** 10.3390/ma14040831

**Published:** 2021-02-09

**Authors:** Rodica Vladoiu

**Affiliations:** Department of Physics and Electronics, Faculty of Applied Sciences and Engineering, Ovidius University, Mamaia Av. no 124, 900527 Constanţa, Romania; rvladoiu@univ-ovidius.ro

There is a great demand for joining carbon, as well as titanium, with other elements, which ensures high resistance against chemical and corrosive environment attacks, as well as, importantly, better adherence of the carbon and titanium to coated substrates.

For instance, this includes titanium-based composites synthesized by the thermionic vacuum arc (TVA) method on substrates, especially for gear wheels and camshaft coating with a direct application to mechanical components of irrigation pumps. Ti embedded in a carbon (C) matrix could improve adherence of the coated substrates to obtain better hardness and anticorrosive properties. Silver has also been recognized as a well-matched metal which is mechanically and chemically stable. Moreover, in wear conditions, the incorporation of silver (Ag) into titanium (Ti) compounds can change their properties by acting as a solid lubricant [1].

Adhesion can be enhanced by an intermediate titanium (Ti) protective coating between a Pt film and the substrate or including the Ti into the Pt matrix. This is supported by the fact that these two metals exhibit quite similar melting points: T_Ti_ = 1941 K and T_Pt_ = 2041.4 K. At the same time, the state of surfaces in the operating conditions of these components becomes more important due to the development and miniaturization of device components [2].

This Special Issue is also focused on emerging concepts allowing the design of new or improved materials with improved nanostructure performance, as well as the characterization of the microstructure and properties of carbon-based materials with high resistance to heat and wear.

Reduced activation ferritic and martensitic steels, like EUROFER (9Cr-1W), are considered as potential structural materials for the first wall of the future next-generation demonstration power station (DEMO) fusion reactor and as a reference material for the International Thermonuclear Experimental Reactor (ITER) test blanket module. The primary motivation of this work is to study the re-deposition of the main constituent materials of EUROFER, namely, tungsten (W), iron (Fe), and chromium (Cr), in a DEMO-type reactor by producing and analyzing complex W_x_Cr_y_Fe_1-x-y_ layers produced by the same thermionic vacuum arc technology mentioned before [3].

In the same toroidal magnetic fusion experiments, the behavior, the properties, and the resilience of the ultra-nano-crystalline diamond (UNCD)-coated probe casings were reported when they were used in the still rather hot and dense edge plasma of the Experimental Advanced Superconducting Tokamak (EAST). The results confirm that the UNCD coating also almost completely prevented the sputtering of graphite from the probe casings and thereby the subsequent risk of re-deposition on the boron nitride isolations between probe pins and probe casings by a layer of conductive graphite [4].

This Special Issue covers subjects concerning the characterization and applications of the nanostructured complex combination, including new technology trends and applications. In this way, organometallic compounds (carbon-based materials with a heavy metal ion in the molecule) mainly coordinate ligands through carbon–metal covalent bonding, leading to phosphorescent processes that are very useful in electroluminescent devices. The organometallic compounds reported a significant *trans*-effect of Ir-C, which induces the formation of Ir-Cl bridge bonds *trans* to the Ir-C bonds. This trans effect leads to an isomer with the C- and N-donor atoms having a *trans* position to each other. The most efficient organometallic compounds are formed from ortho-metalated carbon-based ligands with superior properties compared with ancillary carbon-based ligands [5]. Another combination of elements has been investigated, and the prospects of iron oxide films and their sulfidation for dye-sensitized solar cells (DSSCs) were reviewed. Iron oxide thin films were prepared by hollow cathode plasma jet (HCPJ) sputtering, with an admixture of oxygen in the argon working gas and with an iron nozzle as the sputtering target [6].

On the other hand, the research on improving the substrate influence as well as the sample preparation has been taken into account, and applied to the hydration resistance of CaO refractories. Both Ti and Al chelating compounds enhanced the hydration resistance of the CaO material significantly, making the surface structure much denser. Chelating compounds decomposed at a high temperature, and the decomposition products reacted with CaO to form water-resistant compounds which filled the pores and cracks located on the surface of the CaO material. Comparing the experimental results, it can be stated that the pretreatment with Al chelating compound on the surface of the CaO sample improved the hydration resistance of the CaO material tremendously [7].
